# Impact of developmental language disorders on mental health and well-being across the lifespan: a qualitative study including the perspectives of UK adults with DLD and Australian speech-language therapists

**DOI:** 10.1136/bmjopen-2024-087532

**Published:** 2024-10-22

**Authors:** Adrienne Wilmot, Mark Boyes, Rachel Sievers, Suze Leitão, Courtenay Norbury

**Affiliations:** 1 Curtin School of Population Health and Curtin enAble Institute, Faculty of Health Sciences, Curtin University, Perth, Western Australia, Australia; 2 Independant Speech and Language Therapist, Brighton, UK; 3 Curtin enAble Institute, Curtin University, Perth, Western Australia, Australia; 4 Curtin School of Allied Health, Curtin University, Perth, Western Australia, Australia; 5 Psychology and Language Sciences, University College London, London, UK; 6 Special Needs Education, University of Oslo, Oslo, Norway

**Keywords:** mental health, public health, qualitative research

## Abstract

**Abstract:**

**Objective:**

This study aims to explore the educational, occupational and socioemotional experiences of people with developmental language disorder (DLD) across the lifespan to gain insight into risk and protective factors for mental health.

**Design:**

Qualitative analysis of focus groups and written submissions. Data were combined and analysed using Braun and Clarke’s reflexive thematic analysis approach within a critical realist framework.

**Setting:**

Southeast England and Western Australia.

**Participants:**

Six adults with DLD from England and five speech-language therapists from Western Australia participated in focus groups and/or contributed written responses to the research team.

**Results:**

We developed four themes: ‘perspectives on diagnosis and living with an invisible disability’ explores participants’ perspectives on diagnosis, the difficulty getting a diagnosis and perceptions of DLD as widely misunderstood; ‘school struggles and self-esteem from past to present’ details school experiences and their impact on mental health; ‘DLD across the lifespan’ explores DLD in adulthood with a focus on workplace difficulties, emotional well-being and mental health; ‘A sense of belonging: communication, connection and support’ provides a lived experience account into the social participation difficulties of adults with DLD and the importance of social support.

**Conclusions and implications:**

Adults with DLD may experience poor self-esteem, anxiety and depression. These mental health concerns may result from (a) exhaustion due to masking/compensating for neurodevelopmental differences, (b) loneliness and/or disempowerment due to difficulties with social interaction and (c) adverse experiences such as bullying, discrimination and a lack of appropriate accommodation at school and in the workplace. A lack of awareness and support for people with DLD from health, education and employment providers was also seen as a contributing factor to poor mental health. Diagnosis may serve a protective function for mental health via self-understanding, self-esteem and self-advocacy.

**STRENGTHS AND LIMITATIONS OF THIS STUDY**

- This is one of few studies to recruit adults with developmental language disorder (DLD) to share their perspectives on the educational, occupational and socioemotional experiences of living with DLD.
- Adult participants with DLD contributed to the development of the study’s themes and conclusions.
- Adults with DLD were provided with a range of methods to engage with the research questions (eg, written or oral submission) depending on their communication preferences.
- Our sample size was small, reflecting the fact that few adults with DLD have been formally diagnosed and the nature of language and literacy challenges presents barriers to taking part in research.
- In seeking out a support group for DLD, our group of adults may not be representative of the entire population of adults with DLD, many of whom may not be experiencing such significant challenges or may not have pursued a diagnosis; identifying such individuals may be challenging, but future research should endeavour to gather a wider perspective on adult life with DLD.

## Introduction

 Global prevalence estimates indicate that 6%–8% of children experience unexplained difficulty with language (ie, both using and understanding language); a figure that equates to approximately two children in every classroom.^1^,^3^ An individual can be diagnosed with (developmental) language disorder (DLD) if language difficulties are persistent and confer significant functional impact in the absence of a differentiating condition (such as autism or intellectual disability) in which language disorder may also occur.^4^ DLD is the diagnostic term agreed by international consensus that supersedes the research diagnosis of ‘specific language impairment’.^5^ Once considered a childhood condition, there is increasing awareness that DLD persists into adulthood^6 7^ and is associated with increased risk for adverse outcomes in employment, well-being and societal participation.^8^ However, research exploring the lived experiences of adults with DLD is sparse. This study aimed to explore the educational, occupational and socioemotional experiences of people with DLD across the lifespan to gain insight into risk/protective factors for mental health. Such perspectives are needed to identify research and advocacy priorities and inform the timing and focus of interventions to support the health and well-being of people with DLD.

### Education, employment and mental health

DLD is one of the most prevalent neurodevelopmental conditions yet remains one of the most poorly funded and least researched.^9^,^11^ Nevertheless, there is burgeoning evidence to suggest that children with DLD are at elevated risk of experiencing academic, emotional and behavioural difficulties^2 12 13^ which often persist into adulthood.^14 15^ Links between childhood language difficulties and poor literacy are particularly well established^7^ and contribute to school struggles, for example, poorer academic self-esteem and poorer academic outcomes.^8 16^ Importantly, emotional difficulties (ie, anxiety and depression) among children, adolescents and young adults with DLD do not appear to be explained by language severity or cognitive factors ^17 18^ implying that the association between DLD and mental health is influenced by potential mediating and/or moderating factors. Consistent with this view, there is strong evidence that depression among adolescents with DLD is influenced by their educational and occupational experiences^18 19^ and that young adults with DLD, relative to their peers, experience more difficulty in the job search process, longer periods of unemployment and are more likely to be engaged in lower paid work.^8^ Taken together, these findings suggest that adults with DLD may need ongoing support, especially at times of educational and/or employment transition.

### Socioemotional skills, peer problems and mental health

Given theoretical links between language and socioemotional learning (eg, social cognition, emotional awareness^20 21^), exploring the social experiences of children and adolescents with language difficulties and DLD has been a focus of much past research. Although there is considerable heterogeneity, researchers have found that children with language disorders are more likely to experience challenges with social cognition^22^ and may be considered shy and socially withdrawn.^23 24^ Furthermore, difficulties with social interaction that arise from language limitations frequently manifest as peer problems, in which young people with language disorders are more likely to be excluded by peers, engage in less prosocial behaviour, experience bullying and have poorer quality friendships, leading to increased rates of loneliness.^25 26^ In the general population, developmental changes in loneliness are influenced by language, with higher vocabulary scores in early childhood predicting smaller increases in loneliness over time.^27^ In their longitudinal study, Durkin *et al*^28^ also found that young people with a history of language difficulty entered adulthood with reduced social confidence.

In terms of mental health, peer problems and socioemotional skill difficulties (eg, emotion regulation difficulties) can contribute to emotional difficulties (eg, anxiety) among children and adolescents with DLD.^1929^,^34^ Research investigating associations between socioemotional skills, social experiences and mental health among adults with DLD is underexplored and warrants attention. It is possible, for example, that social difficulties, such as bullying, continue into adulthood and/or that adults with DLD continue to have difficulties with identifying and managing emotions, which may be linked to mental health concerns. In line with this hypothesis, alexithymia, an inability to recognise or describe one’s own emotions, is more common in DLD^35^ and may persist into adulthood.^36^

### Lived experience perspectives

Recently, researchers have turned to qualitative approaches to seek the perspectives of children and adolescents with DLD and/or their caregivers, teachers and clinicians regarding their social and educational experiences^37^,^39^ and mental health.^40^,^42^ Depending on the approach chosen, qualitative methods have the advantage of being able to ground findings in the lived experience of those with DLD without the restriction of standardised measures predetermined by the priorities of researchers. Employing qualitative methods has provided new insight into the nature of language and social difficulties, as well as the risk and protective factors for mental health in the context of DLD. For example, Ekstrom^37^ found that contextual factors at school (such as perceptions of the attitudes of teachers/peers towards them and their DLD) interact with language difficulties to hinder or facilitate communication, class participation and emotional development for young people with DLD. Parents have described how their children’s anxiety can manifest in restricted behaviours and fear of change and intolerance of uncertainty.^40 43^ Others highlight children’s exhaustion as being a maintaining factor for anxiety^40 41^ either due to the effort of camouflaging or masking their language challenges^41^ or the difficulty of meeting the daily demands of school.^40^ Taken together, findings from these studies provide a more nuanced account of socioemotional difficulties among children with DLD, and importantly, of the enormous barriers to accessing support many young people face. For example, in terms of mental health, Hancock *et al*^42^ found that there were a variety of perceived barriers to the identification and support of mental health concerns among children (eg, the inaccessibility of talking therapies) with language disorders.

Currently, only three qualitative studies have included adults with DLD. Myers *et al*^44^ included only young adults (age range 13–23 years), another provided a case study of one woman with DLD,^45^ and the third^40^ focused on adult’s reflections on their childhood experiences. The aim of the current study was to address this gap in the literature by exploring the perspectives of adults with DLD, as well as speech-language therapists (SLTs) who work with young people with DLD, on the educational, occupational and socioemotional experiences of people with DLD across the lifespan to gain insight into risk/protective factors for mental health. Such research is needed to tailor support and interventions for people with DLD as they transition to adulthood.

## Methods

This study is part of a larger project that aims to seek the perspectives and priorities of people with language difficulties, their caregivers and clinicians about emotional well-being and mental health.

### Researcher positionality

We are a group of researchers and clinicians from University College London (CN), Curtin University in Perth, Western Australia (AW, MB and SL) and an independent, UK-based SLT (RS). We include members who have backgrounds in educational and developmental psychology and speech-language therapy and have international collaborations that have given unique insights into DLD across the world. As a research group, we are committed to collaborative research processes to explore mental health among people with language and literacy difficulties. Our past research has explored factors at the individual, family and community level which may influence socioemotional well-being among children with language difficulties in both the written and oral domains^46^ This current study represents our first exploration of mental health among adults with DLD. Notably, the research team includes SLTs from each country, allowing us to explore similarities and differences in service provision and commonalities in the experiences of DLD as reported.

### Materials

We developed two sets of research questions, one for adults with DLD (based in the UK) and one for SLTs (based in Western Australia; see online supplemental materials 1). Both groups had questions which focused on their perspectives about possible links between language disorder and mental health, and research priorities for the field. Adults with DLD were also asked about their experiences of diagnosis, avenues for obtaining health information and support, and perceived support priorities. The research questions were intentionally open ended so that participants could direct the scope and nature of their responses within the broad parameters of the research topic.

### Participants

The sample consisted of six adults with DLD and five SLTs who work with people with DLD and their families. The adults with DLD were either members of a monthly support group for adults with DLD or receiving individual speech-language therapy sessions and recruited via an experienced SLT specialising in adults with DLD (RS, the third author). Some of the adults were known to each other prior to their participation in this research. All but one of the adults with DLD were aged 40–60 years and were currently employed. Two participants mentioned that they had attended a special school for children with communication disorders, while the others had attended mainstream schools during their childhood. Five participants had postschool qualifications. Four of the participants had been diagnosed with DLD in adulthood, for one person this was as recently as 1 year prior to participating in this research. The SLTs were colleagues at the same private practice in a southern suburb of Perth, Western Australia. This practice specialises in working with children and adolescents with language, literacy and other speech and communication difficulties and has an ongoing partnership with the research group. In Australia, SLTs require a 4-year undergraduate or 2-year master-level qualification to qualify to practice. Our sample consisted of SLTs at an early stage of their career (1 SLT: 3.5 years of practice) to a highly experienced SLT (1 SLT: over 40 years of experience).

### Patient and public involvement

This study was part of an ongoing research priority-setting exercise with the DLD community. The focus on the experience of adults and the long-term mental health impacts of DLD has been identified in previous agenda-setting exercises.^9^ Adults with DLD were involved with the development of themes and the conclusions drawn, as described below.

### Procedure

All participants received a study information sheet prior to taking part and were thus fully informed of the nature of participation before providing written consent. The adults with DLD received an ‘easy read’ version of the information sheet adapted by the third author to enhance understanding of the study. They were also offered a variety of means of engagement with the research. At their discretion, they could attend an online workshop, submit a written response to the research questions or have their responses scribed. Participation for the SLTs involved attending a 2-hour workshop facilitated by three members of the research team at their workplace in September 2023. After this, the SLTs were also invited to submit written responses to the research team. All participants were given a gift voucher to thank them for their contribution.

The workshop with the adults with DLD took place in June 2023 and was facilitated by two members of the research team (the second and last author) and the regular support group facilitator (the third author) who was known to the group members. Four adults with DLD attended the workshop, two responded later via written responses to the research questions and three of the workshop attendees submitted written responses in addition to attending the workshop. Both workshops were recorded and artificial intelligence was used to generate a written transcript. After this, the transcript (or relevant sections thereof) was checked against the audio recordings by the first author, corrected where necessary and information which could cumulatively identify any individual was removed and replaced with generic descriptors. At this stage, we decided to integrate the various sources of data (eg, written and oral sources from both adults with DLD and SLTs) for analysis.

After the article had been drafted, all participants were invited to a second workshop (separate workshops for the adults with DLD and the SLTs) to provide feedback on the themes and conclusions. Five adults with DLD and three SLTs attended these workshops and one adult with DLD also provided a written submission. The main findings were endorsed by both groups (adults with DLD and SLTs). However, based on this feedback, we changed the title and focus of theme 1 to incorporate a discussion about diagnosis from the feedback workshop with the adults with DLD. Furthermore, additional quotes (from the adults with DLD feedback sessions) were added across themes.

### Analysis

We chose Braun and Clarke’s^47 48^ reflexive thematic analysis approach to guide our analysis because it is an approach which is both theoretically flexible and suited to analysing heterogeneous data (eg, workshop transcripts, individual written responses).^48^ The first author led the process of coding and analysing the data, seeking the input of the rest of the research team at regular intervals. A critical realist/contextualist framework guided our analysis. This matched our intention to understand the participants perspectives.^48^ For this reason, the data were mostly coded at an inductive (data driven) and semantic level, staying close to the language expressed by the participants during analysis and interpretation. (^48^ p. 56) After coding, an initial set of themes was developed and feedback from other members of the research team was sought (phases 2–3). At the next phase of the analysis, the first author returned to the data to revisit codes and define and refine themes in an iterative process. Direct quotes from participants were selected to represent themes ensuring that different participants from across the dataset were represented within the article (phases 4–6). Each participant is represented in the quotes although there are more quotes from some than others. This is due to variations in how much they contributed verbally during the discussion sessions and whether or not they provided written responses to the questions. Editing of the data extracts consisted of removing some utterances (eg, ‘er’ or word repetitions) and adding punctuation to improve readability. Further editing such as removing irrelevant content or adding explanation to the data is indicated by […]. Written responses have not been corrected for grammar or spelling and are presented as written by the participants.

## Results

We developed four themes from the data which were revised after participant consultation. The first theme ‘perspectives on diagnosis and living with an invisible disability’ explores participants’ perspectives on diagnosis, the difficulty in getting a diagnosis and perceptions of DLD as widely misunderstood. The second theme ‘school struggles and self-esteem from past to present’ explores the impact of school struggles on the self-esteem and mental health of individuals with DLD. The third theme ‘DLD across the lifespan’ explores participants’ experiences of DLD in adulthood with a focus on emotional well-being, and workplace experiences. The fourth theme, ‘A sense of belonging: communication, connection and support’ provides an insider account of the effect of language difficulties on social interaction and the importance of social support.

### Theme 1: perspectives on diagnosis and living with an invisible disability

people can’t see it, think I’m making a mountain out of a molehill (written response, P.01)

DLD, like other neurodevelopmental conditions such as dyslexia and autism, can be considered ‘a hidden disability’.^49^ Without outwardly visible markers, conditions such as these can be masked and also easily misunderstood and/or minimised by other people.^49^ Furthermore, lack of public awareness and professional challenges around diagnosis mean that many people with DLD remain unidentified.^50^ Much of the discussion within this theme focused on the difficulty in gaining a diagnosis, the benefits of diagnosis, and the need to increase community awareness of DLD.

For SLTs, diagnosis meant accurately identifying needs so that they could design evidence-based support, advocate for people at school/college, educate parents and support children’s self-esteem and mental health. However, SLTs did not always find support for diagnosis in the community. For example, one SLT reported that a colleague questioned the value of a DLD diagnosis as it does not often attract government funding for intervention or educational support. There was also a sense that SLTs may face resistance to diagnosis from early years educators who had a ‘wait and see’ attitude. For many of the adults with DLD, having a diagnostic label to describe their difficulties and help identify their needs was linked to improvements in mental health, for example:

I think it is so much worse when your needs are not identified—feeling like your misunderstood, no one understands you, you don’t understand yourself, there is something wrong with you, self-hatred, negative thoughts, self-criticise, self-conscious, believing all the unkind comments and judgement from others, feeling confused / lost / helpless, angry, mixed emotions. (written response, P. 06)

For many, diagnosis was perceived as improving self-understanding, self-esteem and the confidence to self-advocate. In one particularly powerful example, a participant described their recent diagnosis as ‘life-saving’. This participant discussed feeling suicidal, at times, during the years before diagnosis when they were ‘searching, searching’ for answers.

Getting the diagnosis was a lifesaver because I was really at the end. So just thinking, “Well, I can't find out what’s wrong with me”. You know “I'm useless” […] “I can't do anything” [later referring to the way that the diagnosis was explained] she [the speech-language therapist] explained to me, it’s not your fault. You're not stupid, you know, you have a learning disability. You were born with it […] And so that was a life changer for me. So, because of that I started to be, I noticed I start to be kind to myself as well (P.03).

Once diagnosed, many of the adults we spoke to felt that they were better able to advocate for themselves. For example, having a diagnosis and a wallet sized advocacy tool on hand had made a big difference in a recent medical situation for one participant.

That made all the world, that made the big difference, because usually I'd be quiet, wouldn't say anything, and I'd be scared and worried about what’s happening. And then crying at the end of it. But coming away from that experience, being able to tell them, you know, please, you need to tell me again or tell me different words, tell me short sentences. That just really made some, kind of like, the world of difference. (P.03)

Given how important diagnosis was for them, many of the adults we interviewed expressed concern for others with DLD who remain unidentified and/or regret at not being identified earlier. Several participants discussed how getting diagnosed was a difficult process and, in many cases, a matter of chance. For example, one participant had a social worker in the family who was able to advocate for them, another had a SLT in the family who recognised the signs of DLD. Generally speaking, finding information about DLD and avenues for diagnosis and support was perceived as being very difficult. Furthermore, although diagnosis was generally perceived as a step in the right direction, there was a sense that even with a diagnosis, there were still many challenges because many in the community had never heard of DLD. For instance: ‘*It is extremely worse living an invisible disability especially with DLD—that lacks awareness, understanding and SUPPORT! Having to tell and explain DLD to every new person is so exhausting!’ (written response, P.06*).

### Theme 2: school struggles and self-esteem from past to present

Language impacts our ability to learn which affects our mental health and how we view ourselves. (written response, P. 06)

The school years were perceived as a particularly difficult time for many people with DLD for both academic and social reasons. While some participants had felt protected, supported and understood at school, or by particular teachers, others had less favourable memories. Furthermore, many participants linked negative school experiences to problems with self-esteem and mental health. For instance:

I recognised I wasn't doing as well as my peers and took on teacher’s perception that I was lazy, (all reports said I must 'try harder') until I saw a psychologist during my Yr. 11 who helped my MH [mental health]/self-esteem. (written response, P.01.)

Relatedly, school refusal and school-related anxiety among children with language and literacy difficulties were perceived as prevalent by the SLTs. Less commonly, school-related anxiety was linked to suicidal ideation. Another SLT described her frustration at the limits to which she could support children with their self-esteem given a school system that sets them up for failure.

Those students who continuously have difficulty with spelling and writing at school- it can be a ‘kick in the guts’ for them constantly to be marked down […]The system is set up in such a way that those children with language difficulties are destined to fail. (written response, SLT.01)

There was a sense that both social and academic difficulties may worsen in the secondary school environment. For instance, one of the adults with DLD remarked that they had been relatively unaware of their differences in primary school but by secondary school this had changed:

In secondary school I was more aware [of difference to peers] and still received more unkind and hurtful comments—I really wanted to “fit in” or be “normal” (written response, P. 06).

Another related how their academic performance in high school worsened when language demands increased: ‘*I found a transition to secondary school very difficult, and the first year in high school I just went down and down, and all my grades, because I couldn't keep up with the language (P. 01*). This participant was placed in a special class within their secondary school which worsened their self-esteem and mental health.

Transitions from primary to secondary school and from secondary school to post-school options were highlighted by many participants (both adults with DLD and SLTs) as times when intervention and support may be most needed. For example, one participant described their experience of being in supported education as like being in a ‘*bubble’* which didn’t prepare them for the ‘*real world’* when school ended (P. 04). This participant observed that their mental health worsened during their college years without the support they had been used to receiving at school, and that without support at college they had felt ‘*quite alone’* (P.04).’ Another, who had moved away from home to attend college described experiencing anxiety and panic attacks due to being outside the ‘*comfort zone’* (written response, P.06) that their family and home environment had provided.

### Theme 3: DLD across the lifespan

DLD is life-long condition which will affect our mental health throughout our life’s—in higher situations, transitions or points—we need continuous support for both. (written response, P. 06)

All the adults with DLD who participated in our research stressed the importance of recognising DLD as a condition needing understanding, training, funding and support across the lifespan. Although many shared strategies they had learnt over the years to support their mental health, such as sticking to ‘*routines’*, many also expressed continuing difficulties. One adult with DLD stated that daily life with DLD is just about ‘*surviving’* and that ‘*masking’* language difficulties ‘*took up a lot of energy’* and was linked to poor mental health (P.05). Another adult with DLD elaborated on the effort required to get through the day when you have DLD:

Stress, worry, frustration, and daily anxiety trying to; reading information correctly or not, understand what people are saying to you, remembering what people have said, express your thoughts and ideas across and for the other person to understand what you are saying—it is a tiring process and I don’t think people actually understand how much effort we have to go through on daily basis” (written response, P.06).

Two participants referred to having a history of self-harm which one described as a ‘*release’*. Another perceived that mental health concerns may be associated with not ‘*being in touch with my feelings/emotions’ (written response, P.05*). In addition to mental health difficulties, some of the adults with DLD referred to physical health complaints, such as fibromyalgia, which are often stress related. In keeping with this, links between mental and physical health, stress and quality of life among adults with DLD were highlighted as research priorities by the adults with DLD.

Difficulties in the workplace, and links to mental health, were frequently mentioned across our dataset. For many, DLD was linked to difficulties with finding and keeping work, unemployment/underemployment, ‘*struggle to keep up at work’* (written response, P.05), feeling misunderstood or bullied in the workplace, and poverty as a result of employment difficulties. For example, one participant who had experienced a long period of unemployment wrote: ‘*I don’t get passed the interview stage. So, even though I can get reasonable adjustments, it will still be very, very difficult to get a job’ (written response, P. 03*). Another expressed frustration at other people’s perceptions of their capabilities: ‘*I was frustrated by how society viewed the jobs I could apply for (childminding, hairdressing, teachers’ assistant, catering).’ (written response, P. 01*).

There was a sense that workplace experiences were generally better after diagnosis. However, several participants discussed how fear of judgement, bullying and/or concerns about the privacy of their information may prevent them from disclosing their diagnosis in the workplace. Consequently, they may mask their difficulties and get by without support. For instance:

You have to keep plodding along, being miserable and unhappy in the workplace [….] just keep it to yourself, and then going home upset and having to face it the next day because you get accused of being lazy, or you forgot to do this, you forgot to do that. (P.02).

In the workplace and elsewhere, recognition and support for DLD was considered crucial to well-being. For one participant increasing community awareness of DLD was described as foundational; a necessary first step toward the goal of improved mental health: ‘*for DLD we need the awareness first! So everyone is aware of what DLD is, what it means and how to help—to eventually mental health support.’ (written response, P.06*)

### Theme 4: ‘a sense of belonging’: communication, connection and support

Language difficulties disrupt connection/community and then puts individuals more at risk of mental health difficulties. (written response, SLT.04)

In keeping with the child DLD literature,^51^ many of the adults with DLD linked their language difficulties to what they referred to as ‘*social anxiety’*, avoidance of social experiences and loneliness. They described how their language difficulties can result in feeling excluded and difficulty following conversations. For example, one participant stated that they generally understand only about half of what is going on in any conversation and had accepted that ‘*that’s my life!’* (P.02).

There was a sense that novel situations may be more anxiety provoking and exacerbate feelings of disconnection and loneliness. For instance:

Put me in the situation I'm not used to, or I'm fearful, or something like that, I wouldn't be able to kind of like make much sense, and it really makes things worse mental health wise because you get very lonely. (P.03)

In contrast, familiar situations which can be scripted beforehand were generally regarded as less anxiety-provoking. There was a sense among both the adults with DLD and SLTs that expressive and receptive language difficulties can be disempowering. For example, one participant provided the example of ‘*not being able to ask for anything’* for example ‘*the cost of a bus fare’* (*written response, P.05*) as a reason for poor mental health. Furthermore, although participants described situations in which their language difficulties had been accommodated by others (eg, by slowing down speech) they also expressed a perception that other people often find accommodating for DLD difficult. For instance: ‘*People find it hard to accommodate DLD. They can forget, they can patronise, they can be too over-worked to accommodate, or take notice of an individual’s DLD needs.’ (written response, P.01*)

Several participants reported on the importance of having another person in their life for practical and emotional support and to act as a ‘*speaker’* in situations such as medical appointments. Furthermore, having the support of people who understand DLD due to shared lived experience was also highly valued, as expressed in this statement from one participant about the value of the peer support group.

Even though it’s hard to find the money for the group sessions, I still do because it’s as vital to me as eating and gas and electricity because being in a group has a big impact. You don’t feel alone, I learn from other people that they are going through the same thing and just have a sense of belonging. (written response, P. 03)

## Discussion

Six adults with DLD and five SLTs have provided firsthand accounts of the educational, occupational and socioemotional challenges that arise from living with a language disorder. Our participants reported experiencing poor self-esteem, anxiety and depression across the lifespan and highlighted incidents of self-harm and suicidality that have not previously been reported and warrant further investigation. Employment difficulties were a particular concern for many of the adults in our study and were linked to mental health concerns via quality-of-life (eg, worries about financial security), bullying/discrimination, concerns about disclosure and/or exhaustion due to masking/compensating for language difficulties at work. Dyslexic and autistic adults have reported that employment does not necessarily correlate with better mental health in the same way as it does in the general population^52^,^54^ and highlight similar experiences of discrimination, fatigue and working under personal or psychological strain as explanatory factors.^53 54^ Future research into facilitators and barriers to positive employment experiences for adults with DLD is needed if we are to better support the mental health and well-being of adults with DLD.

Poor self-esteem/mental health is rooted in childhood and appears to be influenced by a wider range of school factors, such as difficulty accessing the curriculum and classroom activities, teacher and peer problems, a lack of appropriate accommodations and reasonable adjustments, and a school system that ‘*sets up’* children with language difficulties for failure. Children with DLD may begin to perceive themselves as ‘*stupid’* or ‘*lazy’* in the school context and may camouflage (ie, mask) their difficulties to ‘*fit in’* with peers.^41^ Exhaustion due to camouflaging, in particular, the effort required to compensate for language difficulties during everyday tasks at work, and in other social contexts was also highlighted as a risk factor for mental health. One participant stated that ‘*masking’* their ‘*difference’* took up ‘*a lot of energy’* (*P.05*) and another that having to explain DLD constantly to others was ‘*exhausting’ (P.06*). Researchers have found strong associations between burn-out, camouflaging behaviour and suicidality/mental health concerns among autistic adults^55 56^ and may be risk factors for mental health among children with DLD^40 41^ and children with dyslexia.^57^ Links between camouflaging, exhaustion and mental health among people with DLD across the lifespan require further research.

Our participants indicated that language difficulties may disempower people and/or hinder a natural desire for connectedness to others. For this reason, connection or ‘*belonging’* was highlighted as protective and conversely, the widespread ignorance/misunderstanding of DLD was highlighted as a key risk factor for poor mental health. Communication difficulties may be helped or hindered by social or contextual factors, such as the attitude of the conversational partner, or the novelty of the situation.^37^ For example, having a conversational partner who was willing to slow down their speech, or contextual factors such as having time to prepare speech (ie, scripting), may be empowering and supportive of both communication and mental health. Future research into social and contextual factors that support communication and mental health for people with DLD is urgently needed. Creating more supportive and informed environments for people with DLD to work and learn may lessen their exhaustion and perceived need to camouflage. Future research needs to explore whether greater DLD awareness among health, education and human service professionals improves the accessibility of these services.

Diagnosis of DLD in childhood remains limited^11^ and services for adults with DLD are virtually non-existent. The majority of adults with DLD who participated in our research were positive about receiving a DLD diagnosis, and receiving a diagnosis was perceived to improve self-understanding, self-esteem and self-advocacy skills. Being in a state of knowing that you were different from others but not knowing why was highlighted as a source of distress for some participants. Ideally, diagnosis would be linked to access to greater support but this was not always the case. In keeping with reports from autistic adults,^58^ our participants reported low levels of understanding and awareness of DLD in the community, even among health professionals and teachers who they perceived should understand. In fact, some adults expressed regret that their difficulties had not been identified earlier given the positive role diagnosis played in self-advocacy, which was described as empowering and producing tangible improvements to mental health. Given mixed opinions regarding the value of diagnosis highlighted in this study and elsewhere,^59^ further research exploring the perspectives of children, parents and adults with DLD towards diagnosis and disclosure of diagnosis is needed.

Our study is limited by the small sample size, which reflects the fact that few adults with DLD are formally diagnosed and language and literacy difficulties create barriers to participation in research. Furthermore, in seeking out a support group for DLD, our group of adults may not be representative of the entire population of adults with DLD, many of whom may not be experiencing such significant challenges or may not have pursued a diagnosis. Future research should endeavour to gather a wider perspective on adult life with DLD.

### Conclusion

DLD is an under-researched and underserved condition that persists into adulthood and increases the risk for poor mental health due to (a) exhaustion from masking/compensating for neurodevelopmental differences, (b) loneliness and/or disempowerment due to language difficulties impacting social interaction and (c) adverse experiences such as bullying, discrimination, fear of disclosure and a lack of appropriate accommodation at school and in the workplace. Diagnosis may serve a protective function for mental health via increasing self-understanding, self-esteem and self-advocacy. In addition, increased awareness and support for individuals with DLD are essential foundations for well-being. Future research should endeavour to gather a wider perspective on adult life with DLD and evaluate the impact of interventions across the lifespan on participation and quality of life.

## supplementary material

10.1136/bmjopen-2024-087532 online supplemental file 1

## Data Availability

Data are available on reasonable request.
