# Supplementary material for: Impact of developmental language disorders on mental health and well-being across the lifespan: a qualitative study including the perspectives of UK adults with DLD and Australian speech-language therapists
Source: BMJ Open. 2024 Oct 22;14(10):e087532. doi: 10.1136/bmjopen-2024-087532 (PMC11499835; doi:10.1136/bmjopen-2024-087532)
Supplement: online supplemental file 1 [file bmjopen-14-10-s001.pdf]

Supplementary Material 1. Focus group questions

Adults with DLD

1. *How do you get information or advice about mental health or well-being?*
2. *How do you decide what advice to follow if information is conflicting or confusing?*
3. *Why do you think language difficulties are (sometimes) linked with worse mental health outcomes?*
4. *What do you think are the most important issues that should be researched in the area of language difficulties and mental health?*
5. *What is being done well in terms of research/support in this area?*
6. *What aspects of research/support in this area could be improved?*
7. *What information and/or supports/resources are important to you and should be prioritised?*
8. *Are these questions clear?*
9. *What sort of choices should there be for how people respond?*

Speech Language Pathologists

1) *Why do you think language difficulties are linked with worse mental health outcomes?*
2) *What do you think are the most important issues that should be researched in the area of language difficulties and mental health?*
3) *What is being done well in terms of research in this area?*
4) *What aspects of research in this area could be improved?*
5) *What information and/or supports/resources would you like to see prioritised?*
